# Predicting stone composition via machine-learning models trained on intra-operative endoscopic digital images

**DOI:** 10.1186/s12894-023-01396-2

**Published:** 2024-01-03

**Authors:** Guanhua Zhu, Chengbai Li, Yinsheng Guo, Lu Sun, Tao Jin, Ziyue Wang, Shiqing Li, Feng Zhou

**Affiliations:** 1 Department of Urology, The First Affiliated Hospital of Soochow University, 188 Shizi Street, Soochow, 215006 Jiangsu Province China; 2 Department of Urology, Wuxi 9th People’s Hospital Affiliated to Soochow University, 999 Liangxi Road, Wuxi, 214000 Jiangsu Province China; 3 Mingxu Technology Co., Ltd., 1228 Jiangchang Road, Shanghai, 200072 China

**Keywords:** Morpho-constitutional analysis of urinary stones, Automatic recognition, Deep learning, Ureteroscopy, Kidney stones

## Abstract

**Objectives:**

The aim of this study was to use deep learning (DL) of intraoperative images of urinary stones to predict the composition of urinary stones. In this way, the laser frequency and intensity can be adjusted in real time to reduce operation time and surgical trauma.

**Materials and methods:**

A total of 490 patients who underwent holmium laser surgery during the two-year period from March 2021 to March 2023 and had stone analysis results were collected by the stone laboratory. A total of 1658 intraoperative stone images were obtained. The eight stone categories with the highest number of stones were selected by sorting. Single component stones include calcium oxalate monohydrate (W1), calcium oxalate dihydrate (W2), magnesium ammonium phosphate hexahydrate, apatite carbonate (CH) and anhydrous uric acid (U). Mixed stones include W2 + U, W1 + W2 and W1 + CH. All stones have intraoperative videos. More than 20 intraoperative high-resolution images of the stones, including the surface and core of the stones, were available for each patient via FFmpeg command screenshots. The deep convolutional neural network (CNN) ResNet-101 (ResNet, Microsoft) was applied to each image as a multiclass classification model.

**Results:**

The composition prediction rates for each component were as follows: calcium oxalate monohydrate 99% (n = 142), calcium oxalate dihydrate 100% (n = 29), apatite carbonate 100% (n = 131), anhydrous uric acid 98% (n = 57), W1 + W2 100% (n = 82), W1 + CH 100% ( n = 20) and W2 + U 100% (n = 24). The overall weighted recall of the cellular neural network component analysis for the entire cohort was 99%.

**Conclusion:**

This preliminary study suggests that DL is a promising method for identifying urinary stone components from intraoperative endoscopic images. Compared to intraoperative identification of stone components by the human eye, DL can discriminate single and mixed stone components more accurately and quickly. At the same time, based on the training of stone images in vitro, it is closer to the clinical application of stone images in vivo. This technology can be used to identify the composition of stones in real time and to adjust the frequency and energy intensity of the holmium laser in time. The prediction of stone composition can significantly shorten the operation time, improve the efficiency of stone surgery and prevent the risk of postoperative infection.

## Introduction

Urinary stone disease is highly prevalent worldwide, with prevalence rates of 7–13%, 5–9%, and 1–5% in North America, Europe, and Asia, respectively. Due to the high incidence of new and recurrent stones, renal stone disease represents a substantial burden on healthcare expenditure. Ureteroscopy (URS) combined with laser lithotripsy has a high stone-free rate after operation [1]. At present, URS combined with laser lithotripsy has become the main surgical treatment for urinary stones, mainly for ureteral stones with a diameter of 0.6-2 cm, renal stones (including pyelolithiasis and calyceal calculi), and lower-pole stones(stones lying within a lower (inferior) pole calyx) with a diameter of < 2 cm [2–4], At the same time, as different types of lasers are used for lithotripsy [5], the surgeon’s need to optimize the laser frequency setting is becoming more and more urgent.

Studies have shown that in laser lithotripsy for urinary stones, the laser energy required varies with stone composition and size [6, 7]. Stone composition has a direct effect on the efficiency of holmium laser lithotripsy. For the same lithotripsy result, magnesium ammonium phosphate hexohydrate (MAPH) requires the least energy, while W1 requires the most energy [8, 9]. Today, the urologist usually has to manually select the laser setting based on his or her experience in determining the composition and hardness of the stones during the procedure. The accuracy of stone identification by urologists varies from person to person, which directly results in different times for laser lithotripsy and surgery. A longer operation time increases the risk of postoperative infection [10–12]. Daniel C, Elton et al. constructed a model to measure stone volume based on a patient’s preoperative CT combined with deep learning. Meanwhile Igor Sorokin in one of his 2016 studies mentions stone volume as the best predictor of operative time required for retrograde intrarenal surgery for kidney stones. Combining the results of their research, it became possible for a patient’s preoperative CT to be used in conjunction with deep learning to predict stone volume and thus further extrapolate the length of the patient’s surgery time [13, 14]. Similarly, prompt identification the patient’s stone composition during surgery, using the picture displayed by the ureteroscope, could enable the selection of the most suitable and least power lithotripsy method. This, in turn, would significantly improve the effectiveness of the procedure, reduce surgical time and energy consumption, and mitigate potential thermal harm to the ureteral wall caused by elevated local temperatures [15].

Computer vision and deep learning (DL) can provide solutions to these needs. Image classification is a computer vision task where the computer classifies the image, extracts patterns from the image using deep neural networks, and makes predictions based on the patterns [16–18].

To date, several studies have demonstrated the value of DL in identifying pathological features in diseases such as melanoma and diabetic retinopathy [19–21]. With its emergence as a powerful image-based analysis tool, we investigated the use of convolutional neural networks (CNNs) for the detection of several common types of stone components in humans. By discovering predictive models, it is expected that automatic selection of laser frequency and intensity settings based on real-time analysis of stone composition can be achieved during ureteroscopic holmium laser lithotripsy, thereby significantly improving the efficiency of ureteroscopic holmium laser lithotripsy.

Stone morphology analysis is also essential for the etiological diagnosis of stone disease and can be used to develop new postoperative management strategies. For example, proper dietary modifications can help avoid or minimize recurrence of urinary tract stones [22, 23].

## Materials and methods

### Acquisition of data sets

The data set used in this study was collected from the hospital’s stone laboratory from 490 patients who underwent holmium laser lithotripsy and had stone analysis results between March 2021 and March 2023. Procedures included PCNL, transurethral ureteroscopic holmium laser lithotripsy for stone extraction, and transurethral cystoscopic holmium laser lithotripsy for stone extraction. Stone types included calcium oxalate monohydrate, calcium oxalate dihydrate, magnesium ammonium, apatite carbonate, anhydrous uric acid, and several mixed stone types. All stone data are labelled by a specialist physician and machine with information on the composition of the stones.

### Data preprocessing

**Fig. 1:**
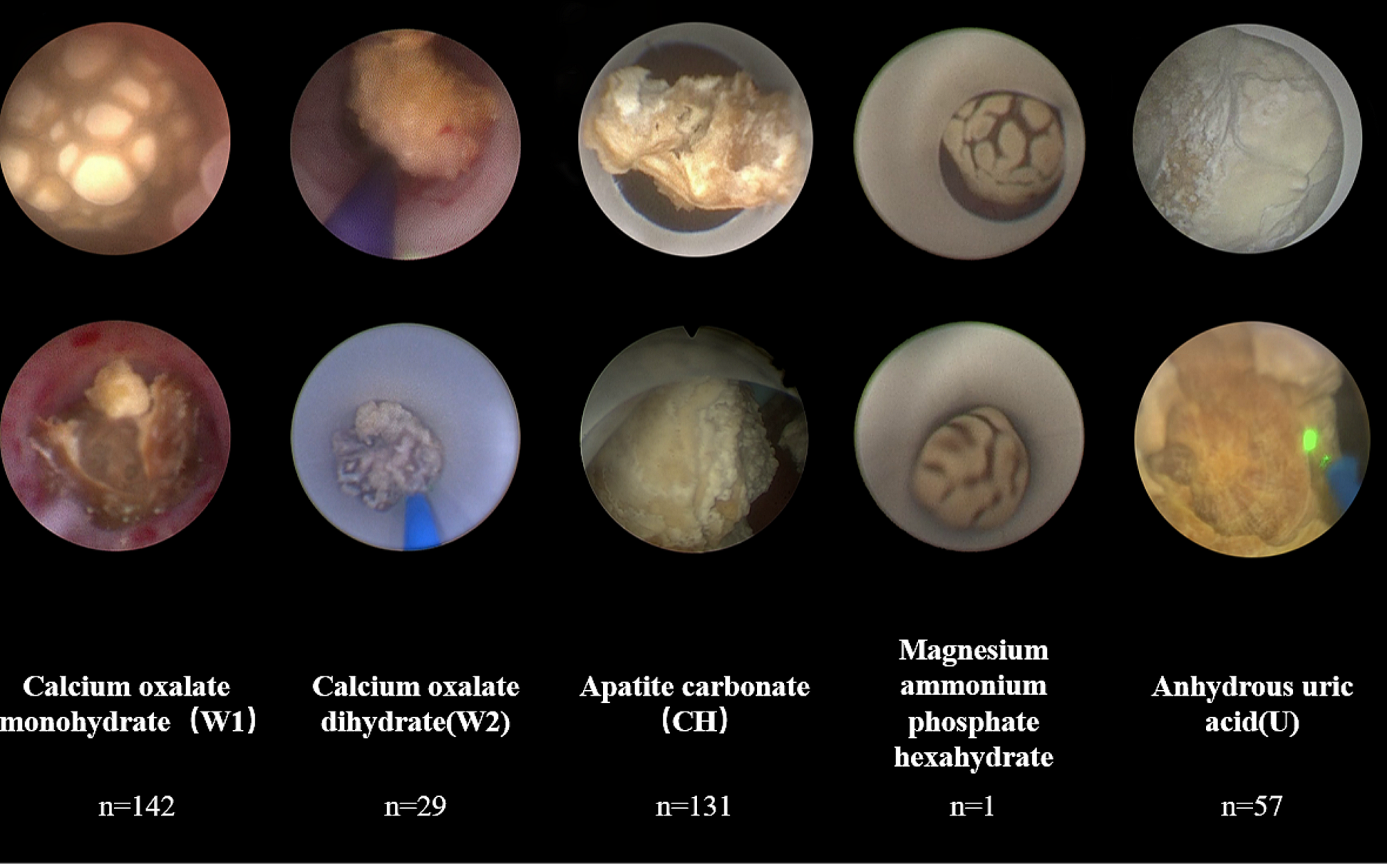
Part of the intraoperative stone images, including the internal and surface images of the stone. (From left to right in the image are calcium oxalate monohydrate, calcium oxalate dihydrate, apatite carbonate, magnesium ammonium phosphate hexahydrate, anhydrous uric acid)

To improve the accuracy of the algorithm, data pre-processing is needed. First, the collected surgical video is converted into a uniform image of size 512*512 by extracting 25 frames per second using the FFmpeg command, as shown in Fig. 1.

After an initial manual screening of images that include the surface and interior of the stone and are clear, the screened images are cropped and scaled to make them uniform in size. Finally, the images were normalized to scale the pixel values between 0 and 1. We classified the stones according to their composition. As the number of mixed stones in the collected stone data was high, the authors uniformly classified mixed stones with a specific stone composition greater than 80% into the pure stone category. If the composition of a particular stone was: calcium oxalate monohydrate composition: calcium oxalate dihydrate composition was 9:1, we classified such stones together with calcium oxalate monohydrate pure stones in the calcium oxalate monohydrate stone category.

### Training of algorithmic models

We mainly applied a deep CNN, ResNet-101 (ResNet, Microsoft), as a multiclass classification model for each stone image. ResNet-101 is a deep convolutional neural network with a depth of 101 layers compared to the traditional model, which allows more feature information to be extracted and thus more accurate analysis of stone components. ResNet-101 uses residual connectivity, which can avoid the problems of gradient disappearance and gradient explosion, and improve the training efficiency and accuracy of the model.

The input image is passed through a series of convolutional and pooling layers to extract features at different levels. ResNet-101 uses residual blocks to solve the gradient disappearance problem in deep neural networks. Each residual block contains two convolutional layers and a jump connection, allowing the network to learn the residual features. The output of the last residual block is globally averaged and pooled to convert the feature map into a vector. The output of the global average pooling layer is connected to a fully connected layer for classification or detection tasks. The output of the fully connected layer is normalized by a SoftMax function to obtain a probability distribution for each category. The final output is the prediction result, which is the category to which the image belongs or the detected target image.

Before training the model, the data set must first be divided into a training set and a test set. The model is then trained using the training set, and its parameters are continuously adjusted by a back-propagation algorithm to better fit the data set. Finally, the model is tested on the test set to evaluate its accuracy and generalisation ability. All the stone images used in the training set are different from those used in the test set.

### Stone composition analysis

Once the model has been trained, it can be used to perform compositional analysis on the new stone images. This is done by feeding the stone images into the model, which outputs a probability vector indicating the probability that the image belongs to a different component. Based on the probability vector, the composition of the stones can be determined.

**Fig. 2:**
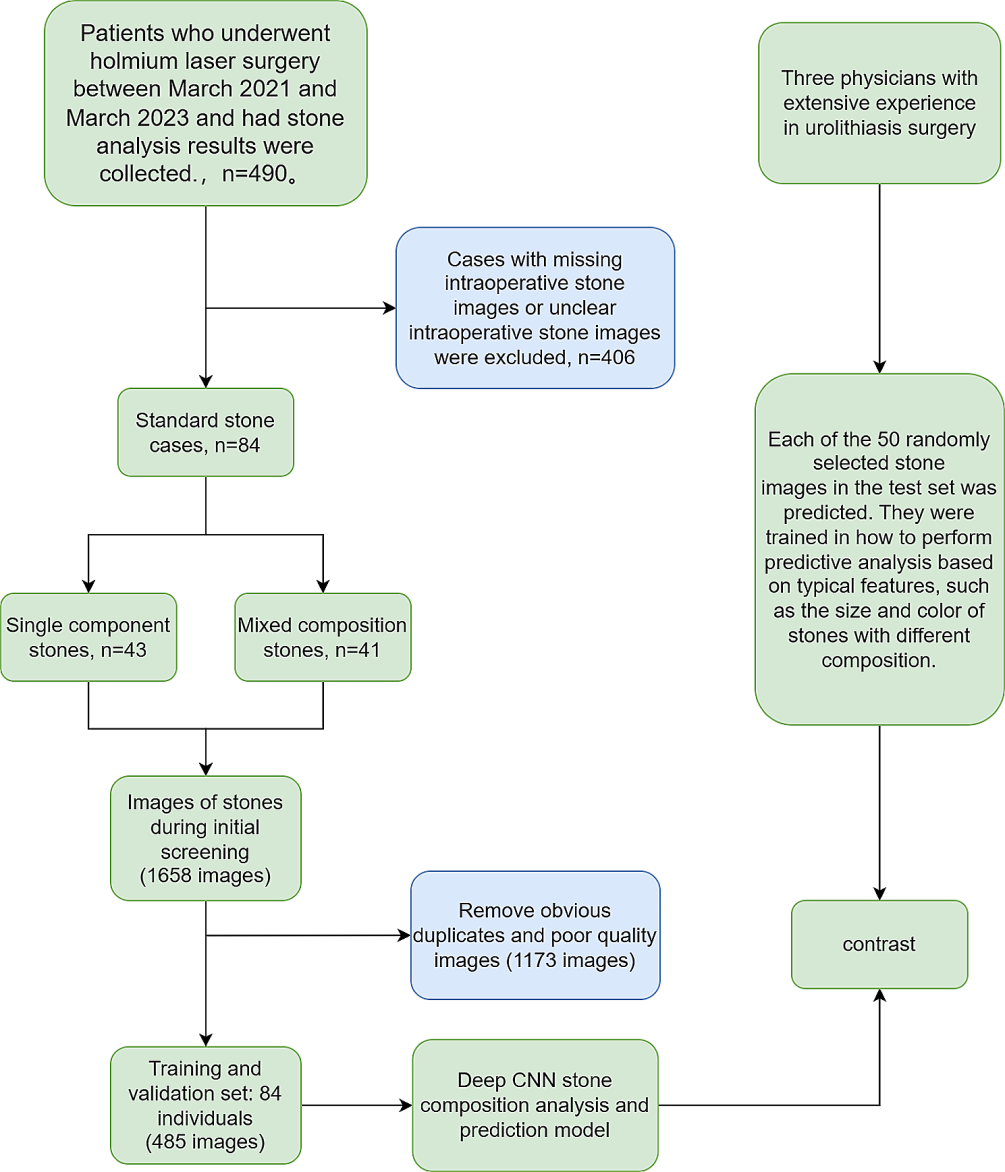
The patients with intraoperative videos were prescreened and divided into two categories according to the composition of the stones, and the blurred images were filtered out. The selected images were then used for training and the training model was compared with clinicians of different seniority. Finally, the prediction accuracy of the two methods was compared

Three doctors with extensive experience in urinary stone surgery (20 years, 9 years and 3 years) were also asked to predict the composition of 50 stone images selected from the test set. They were trained beforehand to perform a predictive analysis based on a number of typical characteristics, such as the size and color of stones of different compositions and the hardness of the stones. The doctors involved in the prediction were only able to see the patient’s stone image data, name, age and date of examination. The performance of our prediction model was assessed by comparing it with that of experienced clinicians, using the results of laboratory stone analysis as the reference standard. (The specific process is shown in Fig. 2)

## Result

A total of 84 intraoperative videos of ureteroscopy holmium laser lithotripsy were selected from 84 patients with stones, including 21 calcium oxalate monohydrate stones, 4 calcium oxalate dihydrate stones, 1 magnesium ammonium phosphate hexahydrate stone, 11 apatite carbonate stones, 6 anhydrous uric acid stones and a total of 41 patients with mixed stones. Of the 1658 images initially screened, 485 images were selected after removing obvious duplicates and poor quality images, including surface and core photographs of single component stones and mixed stones. There were some differences in accuracy between the different stone components, with calcium oxalate dihydrate, apatite carbonate and mixed stones having the highest prediction accuracy, and calcium oxalate monohydrate and anhydrous uric acid stones having slightly lower prediction accuracy.

**Table 1:** Recognition performance measures by stone composition type for ResNet-101 CNN

Stone composition type	Precision	Recall	f1-score	support
W2 + U	1.00	1.00	1.00	24
U	1.00	0.98	0.99	57
W1	0.99	0.99	0.99	142
W1 + W2	1.00	1.00	1.00	82
W1 + CH	1.00	1.00	1.00	20
CH	1.00	1.00	1.00	131
W2	0.97	1.00	0.98	29

**Fig. 3:**
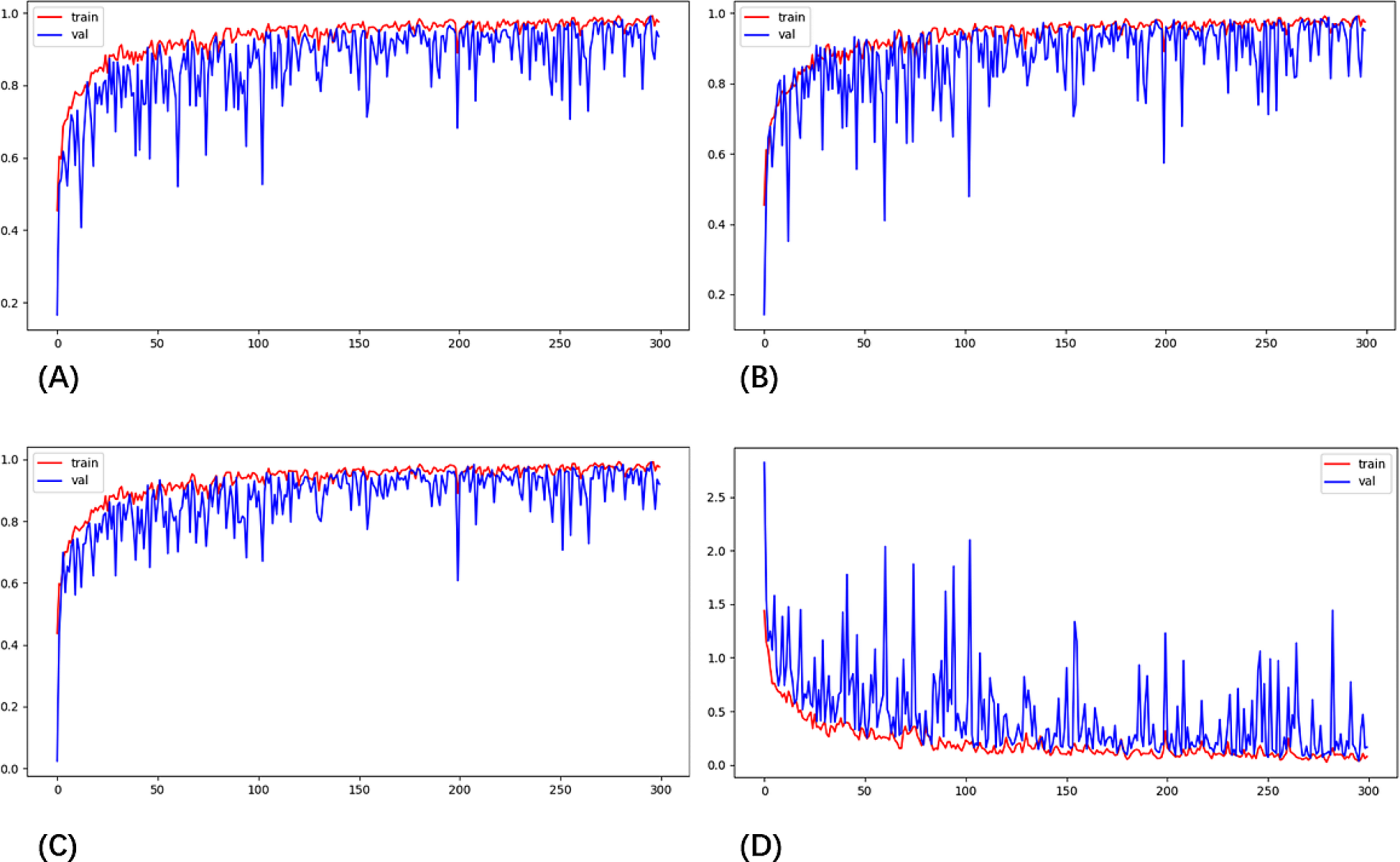
Diagram showing the algorithm (**A**) Accuracy curves. The proportion of the total that the model predicts correctly. (**B**) Recall curves. The proportion of all positive category samples that were correctly identified as positive categories. (**C**) Precision curves. The proportion of positive categories among the samples identified as positive categories. (**D**) Loss curves. The loss curve reflects the distance between the predicted and true values

**Fig. 4:**
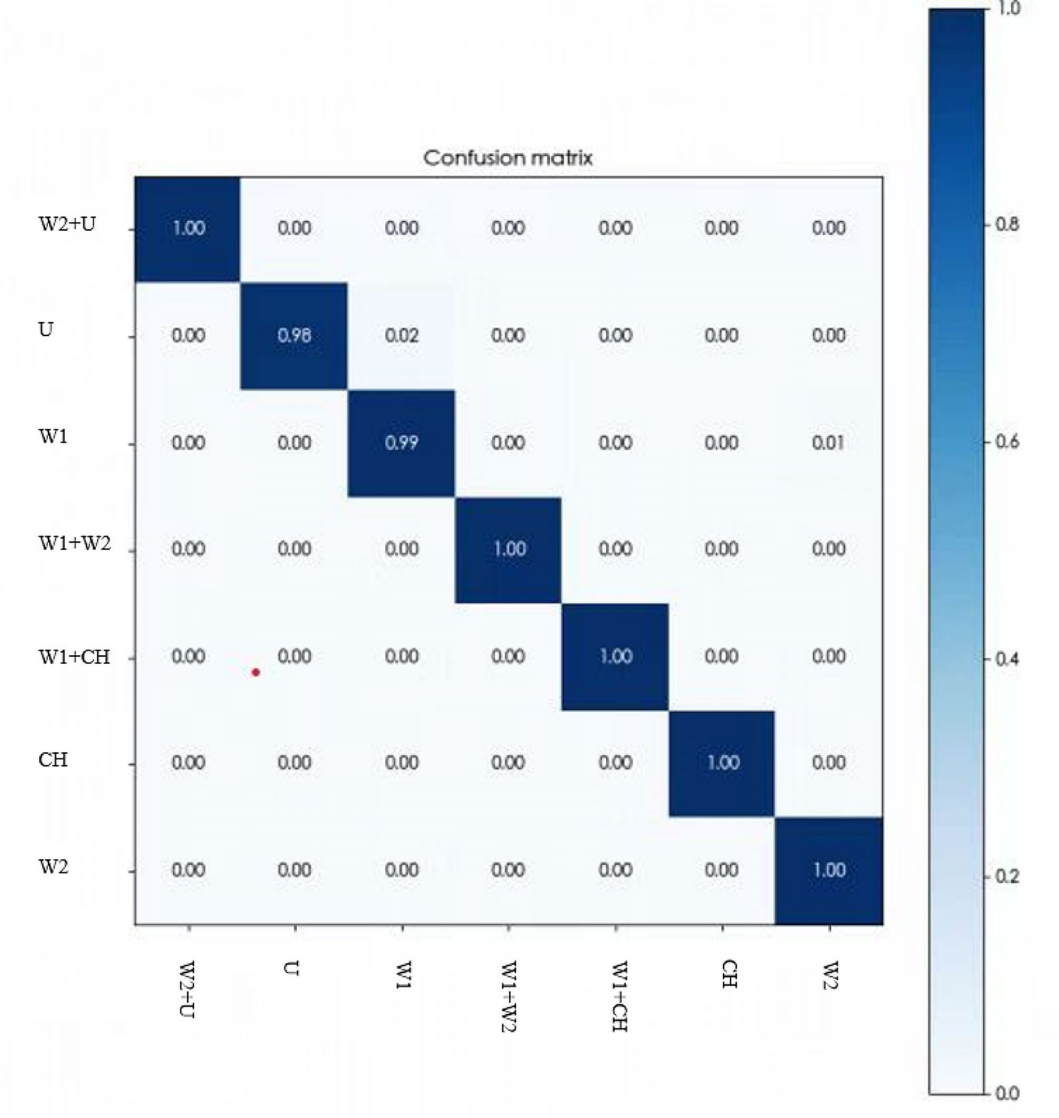
Each column of the matrices represents an actual stone type, while each row represents a predicted type. Dark blue diagonal cells show the percentage of positive predictions by the trained network. Light blue off-diagonal cells correspond to incorrect predictions. The accuracy of the prediction increased as the color deepened

The component prediction rates for each component were as follows: calcium oxalate monohydrate 99% (n = 142), calcium oxalate dihydrate 100% (n = 29), carbonate apatite 100% (n = 131), anhydrous uric acid 98% (n = 57), mixed calcium oxalate monohydrate and calcium oxalate dihydrate stones 100% (n = 82), mixed calcium oxalate monohydrate and carbonate apatite stones 100% (n = 20), mixed calcium oxalate dihydrate and anhydrous uric acid stones 100% (n = 24). The overall weighted recall for cellular neural network component analysis for the entire cohort was 99% (Table 1). The final subject precision-recall curves, and confusion matrices, are shown in Figs. 3 and 4. The training loss plots for each iteration of the cross-validation experiment are shown in Fig. 3 (D). The relative clinician accuracies were 96%, 76% and 40% respectively. Compared to manual empirical identification, the establishment of the AI model significantly improved the accuracy and real-time performance of stone composition prediction. Similarly, there are many studies at home and abroad on the prediction of stone composition from preoperative stone CT values [24, 25], but most of them are limited in the selection of CT values because of the predominance of mixed stones in the clinic. It is not possible to identify the entire stone composition and cannot provide real-time intraoperative feedback on the composition of each part of the stone, which results in the limitation of the application of this technique. However, we believe that through the continuous development of technology, the prediction model by preoperative CT values can also be widely used in the near future because of its accuracy and affordability.

## Discussion

Manual identification of stone composition is known to be a tedious task requiring manual analysis by experienced practitioners. There are many disadvantages to this method, such as the time and effort required, susceptibility to error and inconsistency, and high cost. Recently, it has been suggested that a standard CT scan can be used to non-invasively assess stone composition prior to treatment. The results have shown that CT attenuation values are useful in predicting stone composition, but this parameter has limited value as a predictor of stone composition [26]. The development of advanced techniques that can accurately predict stone composition is essential to enable clinicians to select the most appropriate treatment for patients with stone disease.

In recent years, an increasing number of researchers have started to explore the use of AI technology to achieve real-time stone composition recognition. Compared with manual identification of stone composition, AI technology can complete the identification of stone composition more quickly, reducing the workload and time cost for doctors. Compared to manual identification, which takes minutes or more, AI technology can complete the analysis of stone composition in seconds and with much higher accuracy than manual identification.

AI technology can also improve the accuracy and reliability of stone composition recognition by reducing human interference and training models with large amounts of data. As manual recognition is subjective and inconsistent, the use of AI technology can eliminate these problems, thereby improving the reliability of stone composition recognition. AI technology can reduce the cost of stone composition identification by reducing the physician’s workload and time as it can be done automatically. AI technology enables real-time stone composition identification. Since AI technology can analyze stone composition in seconds, it can improve the real-time identification of stone composition and thus better guide doctors in their treatment. Black K M et al [17] and Estrade V et al [18] used in vitro stone imaging to fully test the possibility of distinguishing stone components according to stone imaging.

This work is more practical than simple in vitro image analysis. By analyzing real-time intraoperative stone images, we attempt to adjust the laser frequency and energy in real-time according to the stone composition automatically identified by AI during holmium laser lithotripsy.

## Conclusion

Based on the analysis of patients’ intraoperative stone images, a deep learning computer vision algorithm based on the ResNet101 model was proposed to predict the composition of urinary stones. Compared with the previous training of an AI recognition model based on in vitro stone images, this experiment directly uses intraoperative stone images, which is closer to the actual application scenario. The trained model is closer to the clinical needs, which can further greatly improve the operation efficiency and reduce the operation time. However, there are also obvious shortcomings in intraoperative real-time images, such as more intraoperative interference factors, bubbles and stone powder will affect the acquisition of real-time images, and it is more difficult to obtain stone components compared with in vitro stone images. It is believed that as the resolution of various endoscopes improves, subsequent identification will become more accurate.

## Data Availability

Not applicable.
